# Risk Factors Associated with Death in In-Hospital Pediatric Convulsive Status Epilepticus

**DOI:** 10.1371/journal.pone.0047474

**Published:** 2012-10-26

**Authors:** Tobias Loddenkemper, Tanvir U. Syed, Sriram Ramgopal, Deepak Gulati, Sikawat Thanaviratananich, Sanjeev V. Kothare, Amer Alshekhlee, Mohamad Z. Koubeissi

**Affiliations:** 1 Department of Neurology, Children's Hospital Boston and Harvard Medical School, Boston, Massachusetts, United States of America; 2 Department of Neurology, Case Western Reserve University and University Hospitals, Cleveland, Ohio, United States of America; 3 Department of Neurology, St Louis University, St. Louis, Missouri, United States of America; 4 Department of Neurology, George Washington University, Washington, D.C., United States of America; University of Utah School of Medicine, United States of America

## Abstract

**Objective:**

To evaluate in-patient mortality and predictors of death associated with convulsive status epilepticus (SE) in a large, multi-center, pediatric cohort.

**Patients and Methods:**

We identified our cohort from the KID Inpatient Database for the years 1997, 2000, 2003 and 2006. We queried the database for convulsive SE, associated diagnoses, and for inpatient death. Univariate logistic testing was used to screen for potential risk factors. These risk factors were then entered into a stepwise backwards conditional multivariable logistic regression procedure. *P-*values less than 0.05 were taken as significant.

**Results:**

We identified 12,365 (5,541 female) patients with convulsive SE aged 0–20 years (mean age 6.2 years, standard deviation 5.5 years, median 5 years) among 14,965,571 pediatric inpatients (0.08%). Of these, 117 died while in the hospital (0.9%). The most frequent additional admission ICD-9 code diagnoses in addition to SE were cerebral palsy, pneumonia, and respiratory failure.

Independent risk factors for death in patients with SE, assessed by multivariate calculation, included near drowning (Odds ratio [OR] 43.2; Confidence Interval [CI] 4.4–426.8), hemorrhagic shock (OR 17.83; CI 6.5–49.1), sepsis (OR 10.14; CI 4.0–25.6), massive aspiration (OR 9.1; CI 1.8–47), mechanical ventilation >96 hours (OR9; 5.6–14.6), transfusion (OR 8.25; CI 4.3–15.8), structural brain lesion (OR7.0; CI 3.1–16), hypoglycemia (OR5.8; CI 1.75–19.2), sepsis with liver failure (OR 14.4; CI 5–41.9), and admission in December (OR3.4; CI 1.6–4.1). African American ethnicity (OR 0.4; CI 0.2–0.8) was associated with a decreased risk of death in SE.

**Conclusion:**

Pediatric convulsive SE occurs in up to 0.08% of pediatric inpatient admissions with a mortality of up to 1%. There appear to be several risk factors that can predict mortality. These may warrant additional monitoring and aggressive management.

## Introduction

Status epilepticus (SE) is characterized by prolonged seizures or by multiple seizures without full restoration of consciousness between events [1]. The condition is associated with significant morbidity and mortality. SE is thought to have a fatality rate of approximately 2% [2], [3]. Complications of SE are also significant and include refractory epilepsy, neurologic deficits and repeated episodes of SE [4].

While a number of studies have identified associations for poor outcomes in SE [5], [6], the use of publically available hospital databases with large sample sizes may allow for better determination of morbidity and mortality risk factors for patients with this condition. In this study, we investigate potential risk factors leading to death in children presenting with generalized convulsive SE.

## Patients and Methods

### Study Population

This study utilized patient data acquired from the Kids' Inpatient Database (KID). The KID dataset has been set up through the Healthcare Cost and Utilization Project (HCUP) and is the only all-payer inpatient care database for children in the United States. Data from both insured and uninsured pediatric patients, defined as individuals less than 20 years of age, are collected. Data collected from KID include demographic information, including patient age, gender, race, median income and ZIP code, primary and secondary diagnoses, procedures, payment information, and patient length of stay. KID also collects information on factors including hospital size, teaching status, type of hospital and hospital location. We used data from the years 1997, 2000, 2003 and 2006. The 1997 database includes data from 2,521 hospitals in 22 states, the 2000 database includes data from 2,784 hospitals in 27 states, the 2003 database includes data from 3,438 hospitals in 36 states, and the 2006 database includes data from 3,739 hospitals in 38 states. Twenty percent of normal newborn births and 80% of the inpatient admissions from each institution are included in the dataset as a systematic random sample [7].

### Standard Protocol Approvals

Prior to data analysis, the Institutional Review Boards of Case Western Reserve University and University Hospitals, Cleveland, OH, and Boston Children's Hospital, Boston, MA, granted exempt status to this study. Informed consent was not required. This study was done in accordance with the HCUP user agreement.

### Data Acquisition

We queried KID for cases with a diagnostic code of generalized convulsive status epilepticus via usage of the International Statistical Classification of Diseases and Related Health Problems, 9^th^ revision (ICD-9) code 345.3. This code corresponds to “grand mal status”. No other ICD-9 codes were used. Data pertaining to month of admission, admission source (from the emergency room, at birth, from outside the hospital and from outside the facility), sex, elective admission or not, age, length of stay, ethnicity, income level (assessed as median income per ZIP code), hospital type (general hospital, children's hospital, or general hospital with a children's unit), hospital bed size, hospital location (rural versus urban and geographic region), and teaching status of hospital were collected. We also used ICD-9 codes to collect data pertaining to patient comorbidities, interventions and complications (table 1). Outcomes pertaining to mortality, which were listed separately in the KID dataset, were collected for each patient.

**Table 1:** Univariate calculations of complications and comorbidities from the KID database.

Predictor	ICD-9 Code	Total N	Predictor N	OR	Lower 95%	Upper 95%	*P*
***Metabolic***							
Hyponatremia	276.1	12360	238	3.31	1.52	7.18	0.002
Hypoxia	799.0, 770.8, 768, 348.1, 768.5, 768.6, 768.9	12360	293	12.35	8.01	19.04	<.001
Drug overdose	966	12360	12	-	-	-	
Hepatic encephalopathy	572.2	12360	<10	-	-	-	-
Metabolic derangements	277, 348.31	12360	105	4.26	1.54	11.76	0.005
Severe malnutrition	260, 261, 262, 263	12360	60	3.65	0.88	15.14	0.074
Toxic	349.82, 323.7, 983, 984, 985, 988, 989	12360	19	-	-	-	-
Hypoglycemia	250.8, 251.0, 251.1, 251.2, 300.19, 775.6	12360	67	8.77	3.46	22.23	<.001
Drug withdrawal	292.0, 779.5	12360	<10	-	-	-	-
***Infectious***							
Sepsis	038, 771.8, 995.91 995.92, 771.81	12360	44	33.56	16.17	69.66	<.001
Cerebral malaria	084.9	12365	<10	-	-	-	-
Disseminated tuberculosis	018	-	-	-	-	-	-
Acute viral encephalitis	049.9, 062, 064, 139.0, 323.0, 323.01	12360	34	10.37	3.12	34.40	<0.001
Central nervous system infections	V12.42	12365	<10	-	-	-	-
Meningoencephalitis	323.0, 323.4	12360	<10	-	-	-	-
Bacterial meningitis	320, 320.7, 320.8, 320.81, 320.82, 320.9	12360	<10	-	-	-	-
Infectious encephalopathy	136.9, 323.5, 323.9, 348.3	12360	394	2.57	1.29	5.10	<.007
Severe malaria	084, 084.9, 084.4, 084.5, 084.6, 084.7, 084.8	12330	-	-	-	-	-
Pneumonia	480, 480.8, 480.9, 481, 482, 482.8, 483, 485, 011.6	12360	51	6.69	2.05	21.78	0.002
Meningitis	013.0, 036.0, 320, 047.8, 047.9, 320.1, 321, 320.8, 322	12360	44	-	-	-	-
Bacteremia	790.7, 771.83	12360	58	-	-	-	-
Viral encephalitis	062, 063, 064, 139.0, 323.0, 049.9	12360	34	10.37	3.12	34.39	<.001
RSV infection	079.6	12360	42	2.57	0.35	18.81	0.354
Postoperative sepsis and liver failure	998.59	12360	26	59.93	26.14	137.42	<.001
Septic shock	785.52, 785.59	12360	46	57.93	30.35	110.59	<.001
***Hemodynamic***							
Congenital heart disease	746.9	12360	<10	-	-	-	-
Hypotension	458, 458.0, 458.1, 458.2, 458.29, 458.8, 458.9	12360	146	15.96	9.28	27.47	<.001
Intractable hypertension	401, 403, 403.0, 405, 405.0	12360	104	5.48	2.19	13.71	<.001
Cardiac failure	428.9	12360	<10	-	-	-	-
Hemorrhagic shock	785.59, 958.4	12360	29	50.93	22.67	114.39	<.001
Post cardiac arrest	997.1	12360	<10	-	-	-	-
***Neurologic***							
Cerebral palsy	333.71, 343, 343.0, 343.1, 343.2, 343.3, 343.8, 343.9	12360	2132	0.64	0.37	1.12	0.118
Subdural Hematoma	432.1, 767.0	12360	21	11.19	2.58	48.59	<0.001
Cerebrovascular accident	434.01, 434.11, 434.91	12360	36	37.07	17.32	82.07	<.007
Brain trauma	850, 767.0, 850.9 854, 310.2	12360	14	-	-	-	-
Acute cerebrovascular disease	436, 437, 437.1, 437.8, 437.9 438, 438.8, 438.9	12360	46	2.34	0.32	17.10	0.403
Brain death	348.8	12360	119	9.19	4.54	18.61	<.001
Brainstem tumor	191.7, 225.9	12360	<10	-	-	-	-
Structural brain lesion	348.8	12360	119	9.19	4.54	18.61	<.001
Intracranial hemorrhage	800.3, 800.8	12360	<10	-	-	-	-
Brainstem herniation	348.4	12360	33	24.46	9.90	60.40	<.001
Epilepsy	345	12360	376	0.84	0.26	2.65	0.763
Generalized Convulsive Epilepsy	345.1	12360	38	-	-	-	-
Generalized Non-Convulsive Epilepsy	345.0	12360	19	-	-	-	-
Infantile spasms	345.6	12360	<10	-	-	-	-
Hydrocephalus	331	12360	832	0.62	0.25	1.51	0.291
Obstructive hydrocephalus	331.4	12360	472	0.89	0.33	2.42	0.821
Communicating hydrocephalus	331.3	12360	16	-	-	-	-
Idiopathic hydrocephalus	331.5	12360	<10	-	-	-	-
Congenital hydrocephalus	742.3	12360	349	0.30	0.04	2.12	0.224
CNS Malformation in fetus	655.0	12360	<10	-	-	-	-
Tuberous sclerosis	759.5	12360	124	0.85	0.12	6.13	0.871
Rasmussen encephalitis	323.81	12360	<10	-	-	-	-
	***Other***						
Non compliance	V15.81	12360	<10	-	-	-	-
Near drowning	994.1	12360	<10	26.38	2.93	237.80	0.004
Congenital malformations	V13.6, V13.69	12360	<10	-	-	-	-
Massive aspiration	507, 770.1, 770.18, E879.4	12360	711	2.25	1.28	3.96	0.005
Esophageal tear	530.89	12360	<10	-	-	-	-
Fever	780.6	12360	612	1.04	0.45	2.37	0.929
**Interventions**							
Transfusion	99.01–99.09	12360	110	36.48	22.21	59.93	<0.001
Ventriculoperitoneal shunt	2.31–2.39	12360	23	4.79	0.64	35.82	0.127
Intubation	96.04	12360	19	-	-	-	-
Mechanical ventilation	96.71	12360	3351	12.64	7.87	20.30	<0.001
Mechanical ventilation 96 hours	96.72	12360	426	22.74	15.53	33.30	<0.001

ICD-9 – International Classification of Disease, 9th edition, RSV – respiratory syncytial virus.

### Statistical Analysis

Univariate logistic testing was used to screen for significant risk factors of death. Risk factors with an associated *p-*value less than 0.20 were entered into a stepwise backwards conditional multivariable logistic regression procedure. The multivariable model only retained risk factors with an associated *p-*value less than 0.05. Two-way interaction terms were entered one at a time into the resulting multivariable model and were retained if the associated *p-*value was less than 0.05. Model validity was assessed using the Hosmer and Lemeshow goodness-of-fit test with *p-*value greater than 0.05 indicating adequate model fit of data. All statistical analyses were performed in Stata 11.0 (Statacorp, College Station, TX).

## Results

### Description of Patient Population and Mortality Rate

A total of 14,965,571 patients were included based on the KID datasets from 1997, 2000, 2003 and 2006. Of these, 12,365 patients (0.083%) were diagnosed with convulsive SE, including 5,541 (44.8%) girls. Five patients (<0.0001%) were excluded from the study due to insufficient data for the tested variables. Mean patient age was 6.2±5.5 years (range <1–20 years, median 5 years). One-hundred-and-seventeen patients (0.95%) with convulsive SE died during their inpatient admission. Additional demographic data are provided in table 2.

**Table 2:** Patient Population.

Total patient population	14,965,571
Cases of Convulsive SE (%)	12,365 (0.083%)
Number of Females with SE	5,541 (44.8%)
Mean Age (SD; range)	6.2 (5.5; 0–20)
Deaths in SE Cases	117 patients
Case Fatality Rate	0.95%

Patient Demographics	*patients*	*percent*
Caucasian	4,697	49.11%
African American	1,943	20.35%
Hispanic	2,044	21.37%
Asian or Pacific Islander	275	2.88%
Native American	71	0.74%
Other	531	5.55%

SE – Status epilepticus; SD – standard deviation.

### Risk Factor Analysis

Logistic testing was done to identify potential risk factors for death in convulsive status epilepticus, and those risk factors associated with a *p-*value of 0.20 were entered into a multivariate model. Factors with a *p-*value of less than 0.05 were taken as significant following model verification. Univariate and multivariate calculations are provided in tables 1, 3 and 4.

**Table 3:** Univariate calculations of hospital and demographic data from the KID database.

Predictor	Total N	Predictor N	OR	Lower 95%	Upper 95%	*P*
January Admission	12360	957	1.00			
February Admission	12360	983	1.95	0.67	5.74	0.22
March Admission	12360	992	2.13	0.74	6.16	0.16
April Admission	12360	878	1.97	0.66	5.90	0.23
May Admission	12360	963	1.59	0.52	4.89	0.42
June Admission	12360	934	1.85	0.62	5.54	0.27
July Admission	12360	935	1.44	0.45	4.54	0.54
August Admission	12360	899	2.14	0.73	6.28	0.17
September Admission	12360	883	1.08	0.31	3.75	0.90
October Admission	12360	999	1.73	0.58	5.18	0.33
November Admission	12360	963	0.99	0.29	3.44	0.99
December Admission	12360	1042	4.11	1.55	10.89	0.01
**Admission Source**	121999					
Emergency room		7674	1.00	-	-	-
Birth		2197	1.53	0.94	2.48	0.084
Outside Hospital		2042	2.07	1.32	3.23	0.001
Outside Facility		286	2.46	0.98	6.21	0.055
Weekend Admission	12146	3389	1.36	0.93	2.00	0.114
Female	12360	5537	1.17	0.81	1.69	0.392
Elective Admission	11333	738	1.34	0.67	2.66	0.48
Age in years (if more than 1 year old)	12319	-	1.03	0.99	1.06	0.108
Age in Days (if less than 1 year old)	801	-	1.00	0.99	1.00	0.327
Length of Stay	12360	-	1.03	1.02	1.04	<0.001
**Race**	12360					
White		4697	1.00			
Black		1943	0.49	0.24	1.01	0.053
Hispanic		2044	0.78	0.43	1.41	0.412
Other		3676	1.43	0.95	2.15	0.088
**Urban/Rural by ZIP Code**	7506					
Large		4306	1.00			
Small		2187	1.27	0.76	2.10	0.361
Metropolitan		638	1.21	0.54	2.73	0.639
Non-core		375	1.18	0.42	3.32	0.754
**Income per ZIP Code**	12006					
1^st^ quartile		3076	1.00			
2^nd^ quartile		3133	1.11	0.64	1.92	0.722
3^rd^ quartile		2776	1.53	0.90	2.59	0.115
4^th^ quartile		3012	1.28	0.74	2.19	0.376
**Hospital Type**	11693					
General		4897	1.00			
General with children's unit		4125	1.60	1.01	2.54	0.045
Children's		2671	2.14	1.32	3.44	0.002
**Hospital Bed Size**	11936					
Small		1696	1.00			
Medium		3430	1.20	0.64	2.25	0.563
Large		6810	1.16	0.65	2.07	0.62
Urban Hospital	11936	11265	3.33	0.82	13.51	0.092
**Hospital Region**	12360					
Northeast		2528	1.00			
Midwest		2488	1.39	0.77	2.53	0.273
South		4125	1.13	0.65	1.98	0.669
West		3219	1.54	0.88	2.68	0.13
Teaching Hospital	11936	9156	2.16	1.23	3.79	0.007

**Table 4:** Significant risk factors for death in pediatric patients with convulsive status epilepticus following multivariate analysis.

Predictor	Odds Ratio	*P* Value	Lower 95%	Upper 95%
**African American Race**	0.36	0.009	0.17	0.78
**December Admission**	3.38	<0.001	2.01	5.67
**Admission Source**				
**Emergency Room**	1.00	-	-	-
**Birth**	1.34	0.279	0.79	2.29
**Outside Hospital**	1.58	0.073	0.96	2.60
**Outside Facility**	2.05	0.159	0.75	5.56
**Comorbidities**				
**Sepsis**	10.14	<0.001	4.02	25.57
**Hypoglycemia**	5.79	0.004	1.75	19.16
**Near Drowning**	43.17	0.001	4.37	426.82
**Hemorrhagic Shock**	17.83	0.001	6.47	49.12
**Structural Brain Lesion**	7.01	<0.001	3.07	15.98
**Massive Aspiration**	9.11	0.008	1.77	47.01
**Postoperative Sepsis with Liver Failure**	14.44	<0.001	4.98	41.86
**Procedures**				
**Transfusion**	8.25	<0.001	4.32	15.78
**Mechanical Ventilation**	9.03	<0.001	5.59	14.60
**>72 hours**				

### Risk Factors Based on Demographic Data

African American children were at a significantly lower risk of mortality following an episode of convulsive SE (p = 0.009). Patients in other racial groups did not have an associated increase or decrease in mortality. No association was found between patient age and mortality risk. Household income, calculated as the average income in a ZIP code region, was also not a significant risk factor for death.

### Risk Factors Based on Hospital Admission

Using univariate analysis, cases referred from outside hospitals were associated with a higher rate of mortality, as were Children's Hospitals, Teaching Hospitals, and General Hospitals with a Children's Unit. These findings were not significant in multivariate analysis. Patient length of stay, weekend versus weekday admission, hospital size and geographic region of hospital were not associated with an increased risk of mortality. None of these factors emerged as significant in multivariate analysis.

### Risk Factors Based on Comorbidities

A number of patient comorbidities corresponded to a greater mortality risk in convulsive SE. Sepsis (p<0.001), hypoglycemia (p = 0.004), near-drowning episode (p = 0.001), hemorrhagic shock (p<0.001), structural brain lesions (p<0.001), massive aspiration (p = 0.008), and postoperative sepsis with liver failure (p<0.001) were associated with increased mortality. Though risk factors such as hypertension, subdural hemorrhage, viral encephalitis, and infectious encephalopathy were significant risk factors in univariate analysis, they did not emerge as significant factors following multivariate calculations. Other comorbidities, including a previous diagnosis of epilepsy, antiepileptic medication withdrawal, fever, esophageal tears, post-cardiac arrest status, brainstem tumors, infantile spasms, other encephalitis, and hydrocephalus were not associated with increased mortality.

### Risk Factors Based on Procedures

The need for blood transfusion was associated with greater risk of death (p<0.001). While intubation was not a risk factor for death, mechanical ventilation for more than 96 hours was associated with a higher mortality (p<0.001). Ventriculoperitoneal shunt placement was not associated with higher mortality.

## Discussion

### Summary

This retrospective study utilized the KID dataset to identify a number of risk factors for poor outcome in pediatric patients diagnosed with convulsive status epilepticus. Higher risk was associated with end-of-year hospital admissions, various patient-related comorbidities, blood transfusion and prolonged mechanical ventilation. African American ethnicity was associated with a lower risk of mortality. A review of the literature is presented in table 5.

**Table 5:** Selected historical studies of status epilepticus with salient findings.

Author, Year and Study Design	Follow up	Mortality rate	Predictors/Risk Factors
Aicardi et al, 1970 [12] *Retrospective*	DischargeUnclear	4.2% (10/239)11% (27/239)	Prolonged SE, cerebral disease
Chevrie et al, 1978 [19] *Prospective*	<4 year f/u	21/334 in 1^st^ year	Symptomatic seizures, age <6 months
Dunn et al, 1988 [13] *Prospective*	Discharge	8.24% (8/97)	Severe pre-existing brain damage, meningitis and encephalopathy
Maytal et al, 1989 [41] *Retrospective with prospective follow-up*	13.2 months	7.2% (7/97)	Prolonged SE
DeLorenzo et al, 1992 [14] *Retrospective and prospective*	7 years	2.3% of children25% in adults	Tumor, hematological disease, anoxia, metabolic and congenital malformations
Scholtes et al, 1996 [42] *Retrospective*	Discharge	11.5% (13/112)	Anoxia, presence of >1 complication, insufficient therapy, prolonged duration (>8 hrs)
Logroscino et al, 1997 [6] *Retrospective*	19 years	21% (38/184)	<1 year age, acute illness
Barnard et al, 1999 [22] *Retrospective*	Discharge53 months	9.6% (5/52)15.4% (8/52)	Brain tumors, metabolic disorder, multi-organ failure
Mah et al, 1999 [10] *Retrospective*	5 years	2% SE group1.5% in NSE group	Drug overdose, sepsis, disseminated tuberculosis, congenital heart disease
Waterhouse et al, 1999 [34] *Prospective*	-	17.8% (5.2 in pediatric and 24%)	CNS infection, hypoxia, drug withdrawal, continuous SE
Berg et al, 2001 [9] *Prospective*	4 years	1.6% (10/613)	Neurodegenerative conditions, coexisting medical conditions
Callenbach et al, 2001 [24] *Retrospective*	4 years	1.9% (9/472)	Respiratory insufficiency, RSV infection, aspiration, brain herniation
Kim et al, 2001 [43] *Case series*	4 years	43.5% (10/23)	Acute symptomatic etiology, especially anoxia
Sahin et al, 2001 [4]Retrospective	8 years	31.8% (7/22)	Remote symptomatic and progressive encephalopathy
Tabarki et al, 2001 [44] *Retrospective*	7 years	15.8% (22/139)	Acute symptomatic seizure, progressive encephalopathy
Logroscino G et al 2002 [39] *Retrospective*	Unclear	43%	Prolonged SE (>24 hr), acute symptomatic etiology, myoclonic SE
Ogutu et al, 2002 [45] *Pharmacokinetics and clinical effects measurements*	Discharge	13.1% (5/38)	Intracranial hypertension, intractable convulsions
Sillanpää et al, 2002 [2] *Prospective*	Long follow up	16% (24/150)	Remote symptomatic cause, young patient (<6 yrs), partial seizures, history of febrile seizure
Singhi et al, 2002 [46] *Prospective*	Discharge	25% (10/40)	Early intubation and ventilation, meningoencephalitis, acute hyponatremia, hepatic encephalopathy
Wu et al, 2002 [3] *Retrospective*	Discharge	1.9% (55/2885)	Female sex, older age (>75)
KarasalIhoGlu et al, 2003 [47] *Retrospective*	1 month	7.2% (6/83)	Polypharmacy, discontinuation of AEDs, neuromotor retardation, generalized background abnormality on EEG
Berg et al, 2004 [30] *Prospective*	Unclear	2.1% (13/613)	Neurodegenerative disorder ± epileptic encephalopathy, prolonged SE
Chin et al, 2004 [26] *Retrospective*	Discharge	5.1% (5/98)	Younger age, intubation, CNS infection, hyponatremia, hypoxia, sepsis, subdural hematoma
Asadi-Pooya et al, 2005 [48] *Retrospective*	Discharge	10.4% (14/135)	Prolonged febrile seizure, CNS infection, metabolic/AED withdrawal, symptomatic epilepsy, prolonged stay
Brevoord et al, 2005 [25] *Retrospective*	Discharge	5.7% (7/205)	Near drowning episode, pneumococcal meningitis, cardiac failure, brainstem tumor, hemorrhagic shock, metabolic defect
Gulati et al, 2005 [8] *Retrospective*	Discharge	30% (9/30)	Prolonged SE (>45 min), septic shock
Kang et al, 2005 [23] *Retrospective*	Unclear	3%	Higher mortality in acute symptomatic SE versus remote symptomatic SE
Maegaki et al, 2005 [49] *Retrospective*	Discharge	3.8% (9/241)	Prolonged SE (>2 hr), moderate-severe asthma
Ozdemir et al, 2005 [11] *Retrospective*	Discharge	19% (5/27)	Acute symptomatic SE, progressive encephalopathy, underlying disease
Ahmad et al, 2006 [50] *Randomized controlled trial*	Discharge	17.5% (N = 160)	Progressive infection, cerebral malaria, febrile convulsions, acute bacterial meningitis, metabolic derangements
Chen et al, 2006 [51] *Population based study*	Discharge	3.1% (7/226)	Febrile illness, acute bacterial meningitis, progressive neurological disorders, intermittent SE.
Morrison et al, 2006 [33] *Retrospective*	Discharge	18% (3/17)	Subdural hematoma, birth asphyxia, post-cardiac arrest
Hayashi et al, 2007 [52] *Retrospective*	Discharge	2.1% (10/479)	Encephalitis, cerebrovascular disease
Muchochi et al, 2007 [53] *Non-randomized controlled*	Discharge	11.54% (3/26)	Pre-existing cerebral malaria, convulsions
Mpimbaza et al, 2008 [54] *Randomized controlled trial*	Discharge	6% (20/330)	Malaria, severe malnutrition, immunosuppression, pneumonia
Sadarangani et al, 2008 [29] *Retrospective*	Discharge	Confirmed convulsive SE: 19% (11/58)Probable convulsive SE: 11% (13/120)	Acute bacterial meningitis, age <1 year, hypoglycemia, focal onset seizures
Siddiqui et al, 2008 [55] *Retrospective*	Discharge	12% (15/125)	Acute intracranial infections, age <5 years, prolonged SE (5.93±5.76 hours)
Lin et al, 2009 [56] *Retrospective*	Stay in ICUUnclear	8.51% (12/141)9.2% (13/141)	Febrile illness
Mei Li et al, 2009 [36] *Retrospective*	1 month	15.8% (32/203)	Mechanical ventilation, complications, SE duration after admission, hyponatremia, recurrent SE
Molinero et al, 2009 [27] *Prospective*	Discharge	13% (6/47)	Infectious cause, cerebrovascular accident, long seizure duration

AED – antiepileptic drug; SE – status epilepticus; CNS – central nervous system; ICU – intensive care unit; RSV – respiratory syncytial virus.

### Mortality rate

The mortality rates of convulsive SE in previous studies vary. Some studies report a less than 2% mortality [2], [3] whereas other studies provide figures over 30% [8]. The mortality rate of convulsive SE in our study was approximately 1%. This finding corresponds to mortality rates from previous research. A state-wide study based in California utilized a dataset including over 19,000 adults and children admitted for SE and found an in-hospital mortality rate of 1.9% [3]. A prospective study evaluating intractable epilepsy in 613 children found a death rate of 1.6% over 4 years [9]. Another prospective study found a mortality rate of 2% in 47 pediatric patients with SE [10]. Studies with higher mortality rates may have analyzed high-risk subgroups, such as SE patients with pediatric intensive care unit (ICU) admissions [8] or cases of refractory SE only [11]. Because prolonged seizures are thought to be a risk factor for complications and death, studies that maintained criteria of a minimum seizure length of 30 or more minutes may have subsequently reported higher fatality rates [12], [13]. Investigators who studied mortality over months to years of follow-up reported higher fatality rates [12]. Most studies of SE do not specifically investigate pediatric patients. Because the mortality of SE may rise with age [5], [14], the inclusion of adult patients can result in a higher case-fatality rate. However, the age-specific incidence of epilepsy is highest in infancy and early childhood and decreases progressively as children grow up [15].

### Demographic data

#### Race

Children of African American ethnicity were found to have lower mortality rates in convulsive SE compared to children of other ethnic groups. In a retrospective study analyzing risk factors for mortality in status epilepticus in Richmond, Virginia, African American ethnicity was found to correlate with decreased mortality risk in univariate, though not multivariate, analysis [14]. While they may have decreased risk of mortality compared to other racial groups, African American children have also been noted to present in SE disproportionately more frequently [3], [16]. Because decreased mortality in status persisted in spite of consideration of socioeconomic factors, a heritable resistance to seizure-related mortality in this population cannot be ruled out.

#### Sex

The importance of gender in the risk assessment of SE is debated. While some studies have identified that males are more likely to present in status [17], other studies have found the opposite [18]. Mortality outcomes in status epilepticus between males and females are similarly conflicting [3], [5], [6]. While our study found that more males presented in SE than females, we did not find sex to be a significant risk factor in predicting mortality outcomes.

#### Age

Some studies have found an association between SE mortality and children of younger ages [19], whereas others have noted that older patients have a higher risk of death during SE [5], [14]. SE is also noted to occur more frequently in younger patients [5]. Age was not found to be a significant independent risk factor for death in our study.

### Hospital Data

#### Time of Year

We tested each month individually as a predictor of mortality and subsequently tested statistically significant months in the multivariable mortality model to account for potential seasonal variations in disease severity, as may occur with infectious or epidemic disease processes. Hospital admissions for convulsive SE during the month of December were associated with an increased risk of mortality. More research will be needed to verify these data and to identify possible causes. Seasonal variations in infections such as bacterial meningitis [20] and viral encephalitis [21] and other respiratory tract infections may have played a role in this annual variation.

#### Teaching and Children's Hospitals

The univariate analysis identified a significant association between deaths and admission into Teaching Hospitals, Children's Hospitals and General Hospitals with a Children's unit. In addition, referrals from outside hospitals are also associated with significant mortality. None of these factors emerged as significant in the multivariate analysis. We believe that some these findings may be related to referral bias, as more severe cases of SE are likely to be referred to Children's Hospitals and teaching institutions.

### Comorbidities

#### Symptomatic epilepsy

Structural brain lesions, as demonstrated by imaging or autopsy findings, emerged as a risk factor for death in our study. This finding is in concordance with results from previous studies suggesting higher mortality in convulsive SE patients with structural lesions, such as brain tumors [6], cerebral dysgenesis [12], [22]_ENREF_13, neurodegenerative disease [6], cerebral palsy [12] or other brain malformations [19]. Mortality may be higher in patients with acute symptomatic SE and neurological injury [23].

#### Pulmonary complications

Aspiration was a common comorbid condition associated with death in pediatric patients with convulsive SE. The risk of aspiration was specifically found to be an independent risk factor for mortality in our study. Pneumonia was identified as a cause of death in a prior retrospective pediatric study [22], a pediatric prospective study [24], and in a pediatric drug trial for management of status epilepticus [25]. In our population it is unclear how many cases of pneumonia were causes or consequences of SE.

#### Sepsis and hemodynamic compromise

Sepsis emerged as an important risk factor for death in our pediatric population. Sepsis may occur as a consequence of pneumonia or from other infective foci. A retrospective study on pediatric ICU patients identified sepsis to be a major cause of death in children with prolonged (>45 minute) episodes of SE [8]. Another study on emergency management procedures in children presenting with SE also identified sepsis as a risk factor for death [26].

Infections may also be an important cause of SE, particularly in cases that were presumably induced by febrile illness or pneumonia [27]. Our study also identified postoperative sepsis with liver failure to be significant enough to constitute an independent risk factor, and this finding is also backed by a previous pediatric case series [28] and by another series on refractory pediatric SE [4]. Hemorrhagic shock was identified as a risk factor for death. In a trial comparing the use of phenytoin and midazolam in school-age children, hemorrhagic shock was noted as a cause of death [25].

#### Metabolic complications

Hypoglycemia emerged as an independent risk factor for death in pediatric convulsive SE. Hypoglycemia increased the risk of neurological sequelae in a prospective study in children [29]. Hypoglycemia may also be a cause of SE [10], [16]. Metabolic complications were associated with an increased risk of mortality using univariate analysis. Previous work has noted similar findings [25] and has more specifically associated deaths to hyponatremia [26]. Liver dysfunction, as mentioned above, is another potential source of metabolic dysfunction.

#### Near drowning

Convulsive SE related to near-drowning episodes constituted an independent risk factor for mortality in our series and other studies [30]. Near-drowning episodes may occur as a consequence of seizures [31]. Conversely, SE may also occur as a result of cerebral anoxia following such an event [32].

#### Intracranial infections

The importance of brain infections in leading to SE or resulting in mortality has been noted in previous studies. A prospective pediatric study identified meningitis and encephalitis as causes of SE. Meningitis was also an important risk factor for death [13]. Similar findings have been noted in prospective [29] and retrospective [26] pediatric studies. Human immunodeficiency virus-associated encephalitis was identified as a cause of SE in one previous pediatric study [4]. In our present data sample, we found intracranial infections, such as viral encephalitis and infectious encephalopathy, to be associated with mortality. These factors did not emerge as independent risk factors for death in multivariate analysis in this series.

#### Other comorbidities

Brain herniation was associated with an increased risk of death. Transtentorial herniation was identified as a long-term cause of death in a prior retrospective pediatric study [24]. Similarly, subdural hematoma, noted as a cause of death in a previous pediatric case series [33], was associated with mortality in our study. However, these factors were not found to be a significant risk factor for death in multivariate analysis. Unlike prior studies [34], patients with status following drug withdrawal did not have a higher mortality rate in this dataset.

### Procedures

#### Blood Transfusion

We identified blood transfusion as a factor for fatalities. This additional risk is probably not a result of the transfusion in itself but is likely a marker for the poor clinical condition of certain patients who subsequently require greater intervention. While this finding was not corroborated by prior research, studies investigating patients with related markers of disease severity, such as those investigating ICU patients, have similarly high death rates [8], [25].

#### Mechanical Ventilation

The need for ventilatory support in convulsive SE may arise from poor respiratory drive resulting from seizure complications or in the context of pharmacological coma induction. The risk of death in mechanical ventilation may be related to the risk of aspiration and impaired mucociliary clearance, leading to pneumonia [35]. Additionally, mechanical ventilation is also associated with complications such as pneumothorax and ventilator-associated lung injury. We confirmed mechanical ventilation as an independent risk factor for death in SE. Mechanical ventilation was associated with death in a prospective study that included adolescents and children [36]. Similar findings were noted in a randomized control trial comparing antiepileptic medications in the treatment of SE [37].

#### Risk factors assessment in SE

We investigated diagnostic entities due to their known association with high risk of mortality. The KID mentions all diagnoses encountered during a hospital admission irrespective of the cause-effect relationship between SE and associated diagnoses. Entities such as near drowning, hemorrhagic shock, structural brain lesion, and hypoglycemia (likely causes of SE), as well as sepsis, prolonged mechanical ventilation, and transfusion (likely resulting from SE or its treatment) increased mortality in children with convulsive SE. Awareness of these risks may thus promote more improved targeted management towards correcting these conditions.

#### Challenges

The findings of this study need to be interpreted in the setting of data acquisition and subsequent analysis. Misclassification of seizures and comorbidities in the ICD-9 coding system is a potential source of error, as it is with all large databases studies. Multiple criteria exist to diagnose SE. While the Working Group of SE of the Epilepsy Foundation of America defines SE as a seizure of at least 30 minutes duration or multiple seizures without full recovery of consciousness in between [1], other investigators advocate a cutoff time as short as five minutes [38]. Individual physicians may not use the same operational definitions, leading to variations in case reporting. The KID dataset lacks information on the timing of procedures, such as blood transfusion and intubation, which may also be important variables in determining the risk of death in SE. Patients who suffer from SE are noted to have an increased risk of dying that extends to weeks, and possibly years, after discharge [39]. This information is unavailable in the KID dataset. Because there are no unique patient identifiers in the KID dataset, it was not possible to account for multiple episodes of SE in the same patient. While neonates were included in this study, there is a lack of consensus on the definition of SE in these patients. This study was unable to investigate certain variables, such as seizure duration, and some etiologies, such as cerebrovascular accidents and malaria, which have been identified as important risk factors in other studies due to few numbers of these cases.

## Conclusion

Convulsive SE is a rare but serious condition and results in approximately 0.1% of all pediatric inpatient hospital admissions. It is fatal in approximately 1% of cases. The rarity of this condition makes it a difficult subject to study. The use of large nationwide databases, such as the KID dataset permits identification of factors associated with increased mortality.

The findings from this study suggest new directions for research. Research should be done to determine which interventions may lead to improved outcomes. More data are needed to ascertain risk factors for long-term mortality in pediatric SE. Parallel research using large groups of adults should be done to establish risk factors for poor outcomes in this group. This may also help to identify risk factors that overlap between pediatric and adult populations. Prospective multicenter studies evaluating children presenting in SE may assist in better determination of risk factors for death and identify those interventions which best promote patient survival.

Our results may also carry implications for improvement of patient care. It is likely that in-hospital mortality of SE is largely a function of the underlying cause of the SE, while other factors may also have an impact on outcome. Some of these risk factors may be ameliorated with specific therapy. Mild induced hypothermia, for example, may be beneficial in patients with near-drowning episodes [40]. Early identification and treatment of some of these aggravating factors may not only prevent immediate mortality, but it may also reduce long-term complications and limit neurological dysfunction. Beyond these implications, the development of treatment paradigms may also help reduce variability in care and outcomes and thereby decrease hospital costs. This research may lead the development of warning algorithms which may in turn promote early interventions that reduce ICU admissions, other medical complications, and even death. Identification of risk factors is thus an important first step towards providing improved care when caring for pediatric patients with SE.
